# Point-of-Care NSE Biosensor for Objective Assessment of Stroke Risk

**DOI:** 10.3390/bios15040264

**Published:** 2025-04-20

**Authors:** Cheng Hsu Chen, Erick Wang, Tsung-Han Lee, Cheng-Chieh Huang, Chun-San Tai, Yan-Ren Lin, Wen-Liang Chen

**Affiliations:** 1 Department of Emergency and Critical Care Medicine, Changhua Christian Hospital, Changhua 50006, Taiwan; 180334@cch.org.tw (C.H.C.); 169118@cch.org.tw (T.-H.L.); 169305@cch.org.tw (C.-C.H.); 2 Department of Biological Science and Technology, National Yang Ming Chiao Tung University, Hsinchu 30010, Taiwan; 3 Industrial Development Graduate Program of College of Engineering and Bioscience, National Yang Ming Chiao Tung University, Hsinchu 30010, Taiwan; 4 AgriTalk Technology Inc., Hsinchu 30010, Taiwan; sandytai@agritalk.com.tw; 5 Department of Post Baccalaureate Medicine, College of Medicine, National Chung Hsing University, Taichung 40227, Taiwan; 6 Department of Nursing, Chang Jung Christian University, Tainan 71101, Taiwan; 7 School of Medicine, Chung Shan Medical University, Taichung 40201, Taiwan; 8 Graduate Degree Program of Biomedical Science and Engineering, National Yang Ming Chiao Tung University, Hsinchu 30010, Taiwan; 9 Center for Regenerative Medicine and Cellular Therapy, National Yang Ming Chiao Tung University, Hsinchu 30010, Taiwan

**Keywords:** neuron-specific enolase, stroke assessment, stroke biomarker

## Abstract

The rapid identification of stroke is critical to improving stroke patient outcomes. Existing protocols for assessing the risk of stroke are subjective and may be further complicated by nonspecific symptoms, increasing the risk of misdiagnosis. Neuron-specific enolase (NSE) has emerged as a promising stroke biomarker. However, current detection methods such as the electrochemiluminescence immunoassay (ECLIA) are time-consuming and costly. In this research, we developed an electrochemical biosensor for the rapid quantification of NSE in whole blood. Mouse stroke models were established, and blood samples collected were analyzed using both hospital-standard ECLIA as well as the biosensor. The biosensor limit of detection was 1.15 ng/mL. NSE measurements were highly correlated between the two methods and were obtained in 5 min using 20 μL of unprocessed whole blood samples. Notably, the biosensor could accurately quantify elevated blood NSE blood that was associated with more severe stroke. Our results demonstrate the utility of the proposed biosensor in pre-hospital settings. Combined with existing stroke assessment methods, the biosensor may enable emergency personnel to identify stroke risk with greater accuracy to optimize the chances of receiving necessary treatment within the effective window.

## 1. Introduction

Acute ischemic stroke (AIS) is caused by a reduction in or blockage of blood flow to the brain and accounts for 85% of all stroke cases. The outcome of AIS patients is highly dependent on timely diagnosis and treatment. Although standard hospital imaging methods such as computed tomography (CT) scans and magnetic resonance imaging (MRI) are valuable tools for accurately diagnosing AIS, they rely on bulky, specialized equipment and trained operators. While mobile stroke units enable CT scans at the point of care, they are prohibitively expensive and have, thus, not been widely adopted. Following diagnosis, the treatment of AIS includes administering a tissue-type plasminogen activator (tPA) to dissolve the blood clot, or performing endovascular thrombectomy (EVT) to mechanically remove occlusions [1,2]. tPA is most effective within 4.5 h of symptom onset [3], while EVT is most effective within 7 h [4]. Patient outcome, therefore, relies heavily on an accurate initial assessment of stroke, enabling emergency medical technicians (EMTs) to deliver the patient to a facility equipped to diagnose and treat stroke within the effective window [5,6,7]. Clinical screening tools such as the Face, Arm, Speech, Time (FAST) test have been established and iterated upon to improve the speed at which EMTs can evaluate the potential of stroke based on patient symptoms [8,9,10]. However, several factors can complicate the initial assessment. In many cases of stroke, patients may present exclusively with non-specific symptoms such as dizziness or low blood sugar that are not limited to stroke [11,12]. Additionally, the interpretation of patient symptoms is highly subjective and results may differ significantly between different pre-hospital medical personnel [13,14]. The inaccurate assessment of stroke occurrence due to inconsistencies in patient symptoms or clinical judgement can greatly delay timely treatment, resulting in preventable disability or death [15]. Therefore, there is an urgent need for more objective methods for assessing the possibility of stroke in pre-hospital settings.

The detection of clinically-relevant stroke biomarkers has been proposed as a more cost-efficient alternative to traditional imaging methods. Neuron-specific enolase (NSE) is an isozyme of the enzyme enolase that regulates glycolysis in neuronal and peripheral neuroendocrine cells [16]. As it is released following neuronal tissue damage, NSE is a valuable marker for identifying potential traumatic brain injuries characteristic of several neurological diseases, including stroke. Blood levels of NSE have been found to be significantly elevated in patients that suffered an acute cerebral infarction [17]. Additionally, an increase in serum NSE is correlated with hypertension, a frequent comorbidity of stroke, and may serve as an early indicator of silent brain damage [18,19]. Moreover, several studies have demonstrated a positive correlation between serum NSE levels at emergency department (ED) admission and both stroke severity [20] and brain infarct volume [21]. NSE may, therefore, serve as a useful objective measure for assessing the extent of acute brain damage. Although NSE is a promising stroke biomarker, its relevance in stroke diagnosis is hampered by the limitations of standard biomarker evaluation techniques. Conventional methods such as ECLIA and ELISA require blood draws and sample preparation and must be performed by trained technicians in a laboratory, and are thus very time-consuming. Delays in biomarker assessment are relevant not only for immediate diagnosis and treatment upon patient admission, but also in the hours following when continuous monitoring could be helpful in predicting the outcome to guide management strategies. Therefore, while blood NSE has been identified as a useful marker in the assessment of stroke severity and prognosis, its practical application in guiding AIS management in actual clinical settings remains limited.

Herein, we describe the design of a biosensor capable of rapidly and accurately quantifying levels of NSE in serum to assist in the diagnosis and management of AIS. Using techniques previously described in [22], we fabricated a label-free biosensor by immobilizing anti-NSE antibodies to gold electrodes for the electrochemical detection of NSE. After verifying the detection of standard NSE, an AIS mouse model was established and serum NSE was measured using both a standard hospital method as well as the developed biosensor. Importantly, biosensor measurements show an extremely high correlation with the standard method while only requiring 20 uL whole blood to achieve detection within 5 min. Our results, therefore, suggest that the proposed biosensor could facilitate accurate and accessible NSE detection to greatly improve patient prognosis when used in pre-hospital settings.

## 2. Materials and Methods

### 2.1. Materials and Apparatus

Hexacyanoferrate (II) trihydrate, potassium hexacyanoferrate (III), isoproterenol (ISO), mercaptoundecanoic acid (11-MUA), N-ethyl-N′-(3-dimethylaminopropyl) car- bodiimide hydrochloride (EDC), N-hydroxysuccinimide (NHS), and isopropyl β-D-1-thi- ogalactopyranoside (IPTG) were purchased from Sigma-Aldrich (Hsinchu, Taiwan). Ni-NTA affinity resin, anti-NSE antibodies, and recombinant mouse NSE protein standards were obtained from BioSmart (Zhubei, Taiwan). Male Balb/c mice were supplied by BioLASCO Taiwan Co. (Hsinchu, Taiwan). Clinical immunoassays were conducted using a Cobas^®^ e 411 analyzer under standard diagnostic protocols. 

### 2.2. Biosensor Fabrication

For protein G expression, GW linker (GINSSSVPGDPPW) was constructed together with a protein G sequence. The resulting biomediator sequence was cloned into the pET-30a(+) vector for recombinant protein expression. *Escherichia coli* (*E. coli*) DH5α was used for plasmid cloning and propagation, while *E*. *coli* BL21 (DE3) was used for protein expression. IPTG was used to induce biomediator expression. For purification, a standard Ni-NTA column was used following a standard purification protocol. Purified biomedia-tor was then used in biochip fabrication.

Biochip fabrication was performed according to previously established methods [22]. Electrodes were immersed in 10 mM 11-MUA dissolved in 99% ethanol for 48 h. The self-assembled monolayer was activated with 100 mM EDC/NHS solution for 1 h. Following activation step, biochips were washed and then immersed in a solution containing 10 μg/mL protein G to allow covalent bonding for 1 h. After protein G was immobilized, chips were washed and blocked with 3% gelatin for 1 h. Finally, biochips were immersed in 1 μg/mL solution of anti-NSE antibodies. Reactions occurred under room temperature.

### 2.3. NSE Detection

For NSE detection, 20 μL of whole blood sample was used to react with the functionalized biochip for 3 min. After binding, the reaction area was washed using 20 mM phosphate buffer (ph 7.4) to remove the unbound molecules. A detection buffer (1× phosphate-buffered saline with 2 mM K3[Fe(CN)6]/K4[Fe(CN)6] (1:1) mixture) was then added to the chip surface. Electrochemical impedance spectroscopy (EIS) was applied to analyze the biosensor chips (frequency: 0.1–1 kHz, amplitude: 10 mV). The impedance data (charge transfer resistance (Rct)) were acquired using the software ZSimpWin (v3.6) in the simulation by the Randles’ equivalent circuit: R(Q(RW)). A standard curve was produced using standard NSE and the formula was input into the software of the device. The impedance data of whole blood sample was interpolated into the formula and the concentration of cTnI NSE was calculated by the software installed in the device.

### 2.4. Establishment of Mouse Stroke Model

Animal experiments were approved by the Animal Care and Use Committee of Changhua Christian Hospital (IACUC approval no. CCH-AE-110-008). Ten-week-old male Balb/c mice were divided into three groups of 10 mice each. Mice in Group A underwent a sham operation without artery ligation. Mice in Groups B and C underwent left-side unilateral common carotid artery ligation for 1 h and 3 h, respectively. A neck incision was made to expose and confirm the left common carotid artery, which was then firmly ligated with 6-0 silk sutures. All mice were adequately anesthetized with isoflurane and analgesic agents throughout the procedures. Following experimental procedures and sample collection, the number of samples suitable for NSE analysis were 3, 6, and 7 mice for groups A, B, and C, respectively.

### 2.5. Assessment of Mouse Stroke Model

After establishing the stroke model, all mice underwent cardiac puncture for blood sampling. The collected blood was divided into two portions. One portion (0.5 mL) was sent to National Yang Ming Chiao Tung University for biochip analysis, while the other (1.5 mL) was sent to a standard clinical laboratory. The blood samples were processed into serum for use in laboratory electrochemiluminescence immunoassay (ECLIA). After sample collection, one mouse from each group was sacrificed to harvest the brain, which was then fixed in 10% formalin solution and sent to the Research Center for Animal Medicine at National Chung Hsing University for histopathological examination. The neuron-specific enolase (NSE) levels obtained from the two methods were compared to validate the performance of the NSE biochip.

### 2.6. Statistical Analysis

Mixed-design ANOVA was used to analyze mice NSE results from both ECLIA as well as the immunosensor. Additionally, due to the small sample size of group C, Student’s *t*-test was used to verify the observed statistical significance. All test values measured by the ECLIA and the biosensor are listed. Graphical regression of R-squared was used for analysis.

## 3. Results

### 3.1. Fabrication of Biosensor Biochips for Detection of Standard NSE

**Figure 1:**
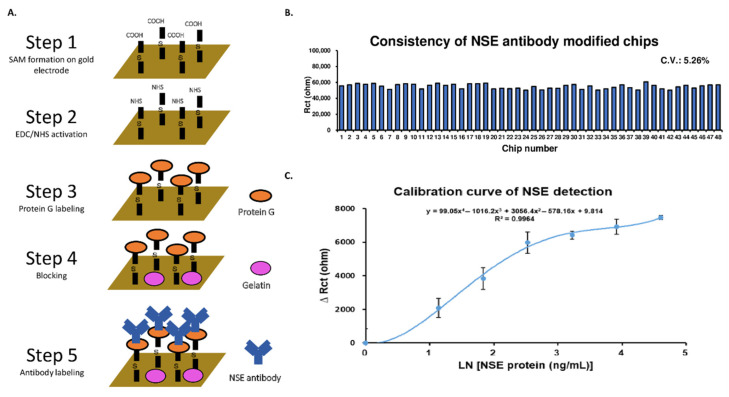
Establishing a biosensor for NSE detection in whole blood samples. (**A**) Biosensor design and fabrication process. (**B**) Verification of functionalized biosensor chip consistency. (**C**) Calibration curve of standard NSE diluted in whole blood samples.

Biochips capable of detecting NSE were fabricated according to the procedure shown in Figure 1A. Following functionalization, the chips were analyzed using EIS to verify modification consistency. Baseline impedance readings of the chips are shown in Figure 1B. The coefficient of variation (CV) of the functionalized chips was 5.26%. Using these chips, a calibration curve of standard NSE spiked in whole blood samples was produced (Figure 1C). The resulting limit of detection (LOD) for NSE was 1.15 ng/mL.

### 3.2. Establishing a Stroke Mouse Model

**Scheme 1:**
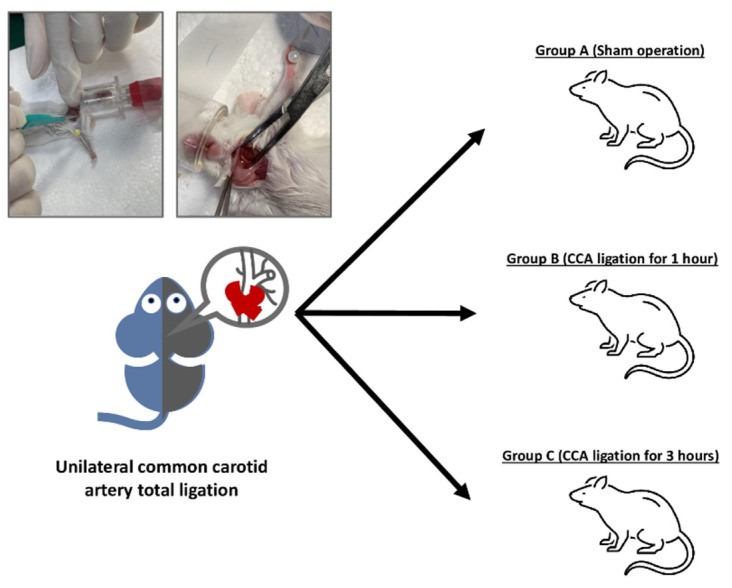
Establishing a stroke model in mice via common carotid artery (CCA) ligation. Mice were separated in Group A (sham operation), Group B (CCA ligation for 1 h), and Group C (CCA ligation for 3 h).

**Figure 2:**
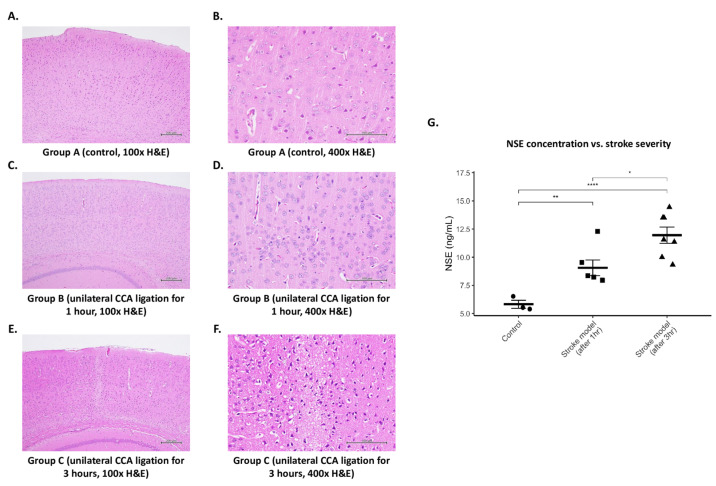
Validation of mouse stroke model. (**A**) Group A brain tissue at 100× magnification. (**B**) Group A tissue at 400× magnification. (**C**) Group B tissue at 100× magnification. (**D**) Group B tissue at 400× magnification. (**E**) Group C tissue at 100× magnification. (**F**) Group C tissue at 400× magnification. (**G**) Measurement of blood NSE levels in three treatment groups using hospital standard ECLIA. Data were analyzed using both mixed-design ANOVA (*p*-value = 0.000495) as well as Student’s *t*-test (* *p* < 0.05, ** *p* < 0.01, **** *p* < 0.0001).

To establish a stroke model that mimics the brain damage caused during stroke, ten-week-old male Balb/c mice were separated into three groups and subjected to different degrees of CCA ligation as depicted in Scheme 1. Group A received a sham operation to serve as control, group B received CCA ligation for 1 h, and group C received CCA ligation for 3 h. The difference in CCA ligation time was meant to model varying degrees of stroke severity that patients might exhibit. To verify the validity of the model, brain tissue from the three groups was observed using hematoxylin and eosin (H&E) staining. The staining results are shown in Figure 2 under both 100× and 400× magnification. Additionally, NSE concentration was measured using the hospital-standard ECLIA (Figure 2G). The results showed a significant difference in NSE level between Group A and Group B, as well as between Group B and Group C.

### 3.3. NSE Detection via Biosensor

**Figure 3:**
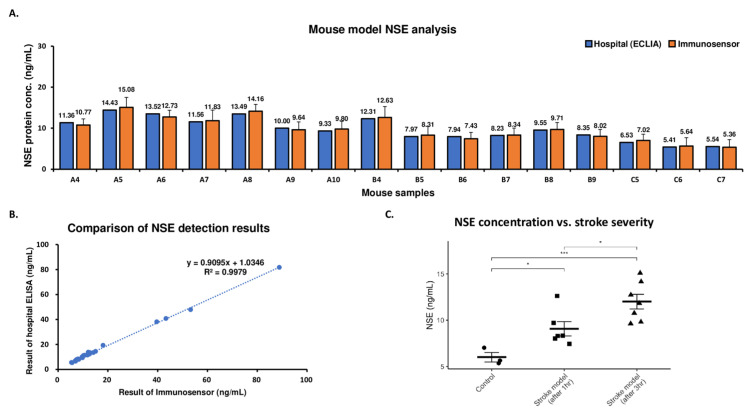
Analysis of NSE in mouse stroke model samples using the immunosensor. (**A**) Detection of NSE in mouse stroke model samples using ECLIA (blue) and biosensor (orange). (**B**) Comparison of hospital standard method and biosensor. (**C**) Concentration of blood NSE in mice from different treatment groups as determined by the biosensor. Data were analyzed using both mixed-design ANOVA (*p*-value = 0.00144) as well as Student’s *t*-test (* *p* < 0.05, *** *p* < 0.001).

Blood samples from mice of all three groups were also analyzed using the proposed biosensor, and the results were compared with the ECLIA measurements (Figure 3A). As shown in Figure 3B, NSE detection using the biosensor correlated very highly with the results obtained using the standard method. Additionally, the increased NSE concentration as a result of increasing brain tissue damage was quantified using the biosensor (Figure 3C).

## 4. Discussion

Identifying stroke risk quickly and accurately remains a challenge in point-of-care settings, as standard guidelines such as FAST rely on a subjective interpretation of patient symptoms. Therefore, an objective measure of biomarkers could greatly assist first responders in more precisely evaluating the risk of stroke.

First, the accurate and reproducible detection of NSE is crucial for enabling emergency personnel to correctly assess the patient’s condition. Previously, we described a method for producing biosensors that possess superior reproducibility, accuracy, and stability suitable for point-of-care testing [22]. Biochips fabricated using this process could detect target protein analytes via EIS rapidly using a small sample volume. In this research, a biosensor was fabricated using anti-NSE antibodies using the same process for stroke evaluation. To verify the consistency of the proposed biosensor, EIS was used to analyze the baseline impedance signals of individual biochips. A CV of 5.26% indicates a highly uniform functionalization of chip surfaces, which allows for the reliable quantification of NSE concentration in blood samples that can be used to guide decision-making in a POC setting.

In order to demonstrate the utility of NSE measurements in evaluating a potential stroke, we established a stroke mouse model via CCA ligation. To assess model validity, brain tissue was observed using H&E staining. Tissue collected from control mice in Group A exhibited a tight, orderly arrangement of neurons with round nuclei and evenly distributed chromatin (Figure 2A,B). In contrast to Group A, tissue from mice that received CCA ligation for either 1 h (Group B) or 3 h (Group C) showed clear pathological changes indicative of neuronal damage. In general, Group C tissue was more eosinophilic compared to control, as shown by the darker staining results. The presence of eosinophilic neurons following ischemic brain damage has been well-documented [23]. At 100× magnification, tissue from Group C (Figure 2E) showed an erratic distribution of neurons in the cortical area as well as blurry boundaries between cells, indicating obvious damage to the tissue structure. At 400× magnification (Figure 2F), significant cavitation can be seen in the cytoplasm, suggesting fluid infiltration due to cell collapse. Additionally, many cells possess shrunken or fragmented nuclei. Pyknosis, followed by karyorrhexis and karyolysis, is a process closely associated with cell necrosis and has been observed in rat neurons subjected to middle artery occlusion [24]. Interestingly, tissue collected from Group B remained structurally intact with regularly arranged neurons at 100× magnification (Figure 2C). However, the observation at 400× magnification (Figure 2D) revealed slight vacuolization in the cytoplasm of some cells. While most nuclei had a normal morphology, some also exhibited nuclear condensation, further suggesting slight ischemic injury. Together, the H&E staining results confirm that treatment with CCA ligation induced a stroke model that recapitulates different levels of stroke severity. Notably, the pathological differences between the three groups mirrored the blood NSE measured using hospital-standard ECLIA (Figure 2G). Specifically, NSE levels were the lowest in the control mice, increased in mice subjected to a 1 h CCA ligation, and rose even further in mice subjected to a 3 h CCA ligation. These observations align with other studies, which have demonstrated a correlation between NSE stroke severity as well as outcome [25]. Therefore, our findings suggest that measuring the NSE level could provide first responders with an objective insight into not only the possibility but also the severity of stroke in patients that could shorten the time to treatment.

Finally, we compared the measured NSE levels using the proposed biosensor and compared the results to the standard method (Figure 3B). The NSE concentrations obtained using the two methods were very highly correlated (R-squared = 0.9979). However, while the biosensor produced similar results to the standard ECLIA test, only 20 μL of whole blood was required for analysis, and detection was completed within 5 min. These results represent a significant improvement in point-of-care applicability when compared to previously proposed stroke biosensors which, while promising, possessed disadvantages such as the longer detection time [26] or additional sample-processing steps [27]. Additionally, a specificity test was performed using S100b, another common stroke biomarker. The biosensor demonstrated a clear, consistent electrochemical signal after binding with NSE but not with S100b, confirming selectivity. Moreover, the biosensor could accurately determine the difference in NSE concentration between mice of different treatment groups (Figure 3C). Crucially, the significant increase in NSE was accurately reflected in the biosensor measurements. These results validate the feasibility of using the NSE concentration as an additional measure to improve the ability to assess the patient’s condition in an emergency situation. While NSE is a strong candidate for use as a stroke biomarker, it is not stroke-specific [28]. However, the biosensor results also demonstrate the possibility of a robust, specific analysis of additional markers such as glial fibrillary acidic protein [29], suggesting the potential of a multiplex stroke panel for even greater diagnostic accuracy.

Minimizing the time needed to accurately diagnose a stroke patient and administer the appropriate treatment is essential to patient survival. However, in many cases, stroke patients may not exhibit typical symptoms, or they may be unable to comply with standard stroke assessment protocols such as FAST. Additionally, the interpretation of patient symptoms is highly subjective and can vary between medical personnel [13]. Combined, these obstacles often lead to inaccurate evaluations and delays in patient treatment. Our previous work demonstrated the possibility of a greatly improving response time to heart attack patients using a biosensor for point-of-care cTnI detection [30]. Here, our results not only confirm the value of NSE as a stroke biomarker, but also demonstrate the potential for a similar reduction in stroke response time. The biosensor possesses several advantages, including a fast detection time (5 min), small sample size (20 μL), and high portability, that allow for a much more rapid quantification of NSE compared to the hospital-standard ECLIA. In pre-hospital settings, the detection of NSE could serve as an objective indicator of stroke severity, thus enabling emergency responders to rapidly determine optimal follow-up actions. For example, unresponsive patients or patients with non-specific symptoms who also exhibit elevated NSE can be transported directly to facilities equipped to treat stroke, thereby eliminating the need transfer to a different hospital should a stroke be identified following arrival. The proposed biosensor could, therefore, be a valuable tool for refining stroke evaluation and improving stroke patient survival when used in conjunction with existing clinical assessment tools such as FAST.

## 5. Conclusions

In this study, we developed a biosensor for the rapid measurement of NSE using whole blood samples. A mouse stroke model representing varying degrees of stroke severity was established using CCA ligation. Blood samples collected from the mice were analyzed using both the biosensor as well as the standard clinical laboratory method. The biosensor demonstrated an LOD of 1.15 ng/mL. A comparison of the results showed a high correlation between the two methods, confirming the viability of rapid and precise NSE quantification. Additionally, higher levels of NSE were observed in mice with more severe ischemic damage. The biosensor accurately reflected varying degrees of stroke within 5 min using 20 μL of whole blood samples. Our results indicate that the proposed biosensor may serve as a valuable tool for objectively assessing the patient’s condition that, when combined with existing protocols, can decrease the time to treatment and improve patient outcome.

## Data Availability

Data are contained within the article.
